# Estimating Rice Cropping Area and Analyzing Land Use and Land Cover Changes in Jiangsu Province Using Multispectral Satellite Imagery

**DOI:** 10.3390/plants15050715

**Published:** 2026-02-27

**Authors:** Kashif Ali Solangi, Canhua Yang, Farheen Solangi, Weirong Zhang, Jinling Zhang, Chuan Jin

**Affiliations:** 1 Key Laboratory of Genetics and Germplasm Innovation of Tropical Special Forest Trees and Ornamental Plants (Ministry of Education), School of Tropical Agriculture and Forestry, Hainan University, Danzhou 571700, China; kashifsolangi@hainanu.edu.cn (K.A.S.); yangcanhua@hainanu.edu.cn (C.Y.); 2 Key Laboratory of Conservation and Development of Hainan National Park, Hainan Institute of National Park, Haikou 570203, China; 3 Research Centre of Fluid Machinery Engineering and Technology, Jiangsu University, Zhenjiang 212013, China; dr.farheensolangi@gmail.com; 4 School of Ecology, Hainan University, Haikou 570228, China; 997350@hainanu.edu.cn

**Keywords:** rice area estimation, Jiangsu province, multispectral satellite data, vegetation index, land surface temperature, land use–land cover change

## Abstract

Climate change and growing populations are major challenges for food security. Understanding single-season rice (SSR) growth patterns and how much area changes over time is essential for sustaining rice distribution patterns and ensuring food security. This study utilized ground trothing data with the remote sensing (RS) technique for estimation of the SSR pattern in Jiangsu Province. A total of 1700 rice and 470 non-rice points were collected during the field visit in April–September 2023 across Jiangsu Province. The current study employed advanced machine learning (ML) and the random forest (RF) model using Google Earth Engine (GEE). This study evaluates the SSR cropping area, including the Normalized Difference Vegetation Index (NDVI), land surface temperature (LST), and land use–land cover (LULC) variation from 2018 to 2023 with different satellites. The results of NDVI show an increasing trend with mean values rising from 0.30 in 2018 to 0.42 in 2023. Additionally, higher mean values of LST were noticed in 2020 by 14.4 °C and in 2022 by 14.1 °C. Furthermore, the SSR area has significantly changed, mostly in the eastern and southern regions of Jiangsu Province, from 2018 to 2023. The higher rice cropping area decreased by 1.42% in 2019 compared to 2018, while the total reduction over the 2018–2023 period was 0.92%. Total cultivated crop areas continued to decline because most of the crop areas changed into built-up areas, resulting in a total variation of 2.75% from 2020 to 2023. The overall accuracy of RF model range was 77.33% to 93.55% with a Kappa coefficient of 0.55 and 0.87, indicating moderate to substantial classification agreement across the study period. The results of LULC indicate that the crop area decreased by 4.13% from 2018 to 2023, and major areas were converted into water bodies and built areas. In conclusion, the single-season cropping pattern decreased during the study period, accompanied by a reduction in total cropland area in Jiangsu Province. Therefore, these findings highlight the influence of urbanization and climate change on agricultural land and emphasize adaptive strategies in Jiangsu Province to ensure food security in the face of environmental challenges.

## 1. Introduction

Globally, rice (*Oryza sativa* L.) is a crucial cereal crop, and it is widely consumed due to its high energy and carbohydrate content [1]. Rice is considered the fourth largest grain crop in the world, and it contributes a total of 8% of food production worldwide. Its total production reached up to 799 million tons in 2023, which grew around 168 million hectares. However, due to the increasing world population, expected demand will increase to 873 million tons by 2030. On the other hand, food security has become a critical issue due to increasing food demands that require widespread attention and discussion [2]. A previous study highlighted that for high-quality rice to meet the high food energy demands of an increasing population, we will need to increase rice production by 31% until 2033 [3].

The rice production in China was > 49.5% rice per hectare, approximately 28% of the total rice farming area, and contributed 19% of the global harvested area [4]. China has two main rice cropping patterns: single-season and double-season. While single-season rice (SSR) crop cultivation occurs once a year, double-season rice cultivation occurs twice a year [5]. Recently, Jiangsu Province has gained more attention for SSR planting due to its ability to maintain soil health conditions. Rapid and unplanned urbanization could have a negative impact on cropping patterns leading to decreased food security [6]. Further, different climatic conditions and environmental factors also significantly influenced rice cropping patterns. Therefore, estimating the rice crop area with novel and reliable methods is very important for future food security. Traditional, only-ground-based methods for estimating rice cropping area are insufficient and are also considered expensive and time-consuming [7].

Therefore, advanced remote sensing (RS) technologies provide an accurate and cost-effective method for performing rice area estimation [8]. These technologies facilitate large-scale examination of rice-producing areas, reducing the costs associated with ground sampling and data acquisition. There are several researchers who used a wide range of various machine learning (ML) models for crop area estimation, including support vector machine (SVM), random forest (RF), and deep learning (DL) [9,10]. Combined use of satellite datasets with ML models has shown promising results in areas of estimation and predicting yield of crops, with the RF model providing best performance [11]. But the performance also largely depends on the representativeness and quality of the data used, so rigorous preprocessing is essential to ensure robust and generalizable learning [12].

The ML approaches offer numerous advantages in crop area estimation and mapping, but it requires the preparation of samples, often in the order of hundreds or thousands, to obtain satisfactory accuracy. An earlier study that used RF procedures to demonstrate accurate maps of wheat and maize yields in China [10]. Other previous studies used Moderate Resolution Imaging Spectroradiometer (MODIS) satellite data at 500 m for rice crop estimation [13] and the Enhanced Vegetation Index (EVI) and the Land Surface Water Index (LSWI) [14,15]. Due to the short flooding period and the cloud effects, some pictures lead to missing the flooding signal and reduced accuracy; this data leads to high image quality demands and resolution time. However, rice cropping pattern images typically use the water index from optical images, such as the LSWI, because flooding conditions during rice planting perform an important role [16]. A previous study proposed that an LSWI value greater than the proposed value of the Normalized Difference Vegetation Index (NDVI) could be utilized to differentiate and classify rice features during the transplanting stage, allowing the extraction of rice area mapping data [17]. Therefore, various large cover areas, such as China, Southeast Asia and Northwest India, adopt this efficient phenological rice area mapping method [18]. Although this efficient phenological mapping method requires time series analysis due to heavy rainfall and cloudy weather, the resolution of MODIS data is coarse [19] and does not extract accurate rice data especially in small plot areas [10]. While synthetic aperture radar (SAR) is able to capture images during the rainy and cloudy conditions [20]. The SAR images provide an alternative method because, unlike optical signals, they can penetrate through clouds and completely avoiding their influence [21]. Numerous studies have demonstrated that SAR can identify rice crops and provide good cloud-free imagery at the regional level [22,23]. On the other hand, the MODIS satellite provides temporal time series data but low resolution (250 m–1000 m), this type of data is commonly used for country or global level. The Sentinel-2 Satellite provides a favorable balance by combining a 5-day revisit time with 10 m spatial resolution in the VIS–NIR bands (and 20 m in the red-edge/SWIR bands). Sentinel-2 application in the agriculture field includes soil moisture monitoring [24], early detection of diseases, pest and abiotic stress [25], and assessment of water and nutrition requirements. Therefore, on the regional scale, since 2015 Sentinel A and 2017 Sentinel l B have provided high-resolution imagery. Thus, in the current study, Sentinel imagery was utilized for SSR estimation and mapping with the RF model. The RF is a machine learning model that provides high accuracy for tree, vegetation, and forest area estimation. For achieving higher accuracy in rice mapping, many ML methods require large amounts of training datasets to obtain optimal model performance [26]. Therefore, in this study, we utilized ground training data with the RF model for estimation of SSR in Jiangsu Province.

Moreover, NDVI is mostly used to estimate the extensive practice of healthy vegetation and its significant interaction with crop productivity [27]. The land surface temperature (LST) serves as an important indicator for determining vegetation health, because LST could effects vegetation health under recent climate variation [28]. Furthermore, land use and land cover (LULC) studies have provided crucial information regarding land use types and understanding cropping patterns, economic demand and sustainable development approaches [29]. Previous studies used a monosatellite source or time series, a single method for time series feature extraction for LULC [23,30,31]. It was noticed that LULC plays an important role in area estimation, as it provides detailed information at regional and global levels. Therefore, these parameters, such as NDVI, LST, and LULC, were used in the current study. The LULC-derived crop data were also compared with SSR statistics, providing reliable and accurate information across Jiangsu Province. The aim of this study was to estimate the SSR of Jiangsu Province using ground truthing data with a machine learning approach to apply the RF model for accurate estimation. While several high-quality remote sensing products and indices are publicly available, few studies have integrated these datasets to specifically quantify and interpret multi-year single-season rice (SSR) dynamics at the provincial scale in China. This study addresses this gap by producing high-resolution SSR maps for Jiangsu Province from 2018 to 2023 and explicitly examining how observed SSR changes relate to land use transitions and urban expansion.

## 2. Results

### 2.1. Spatial–Temporal Analysis Maps of NDVI and LST

**Figure 1:**
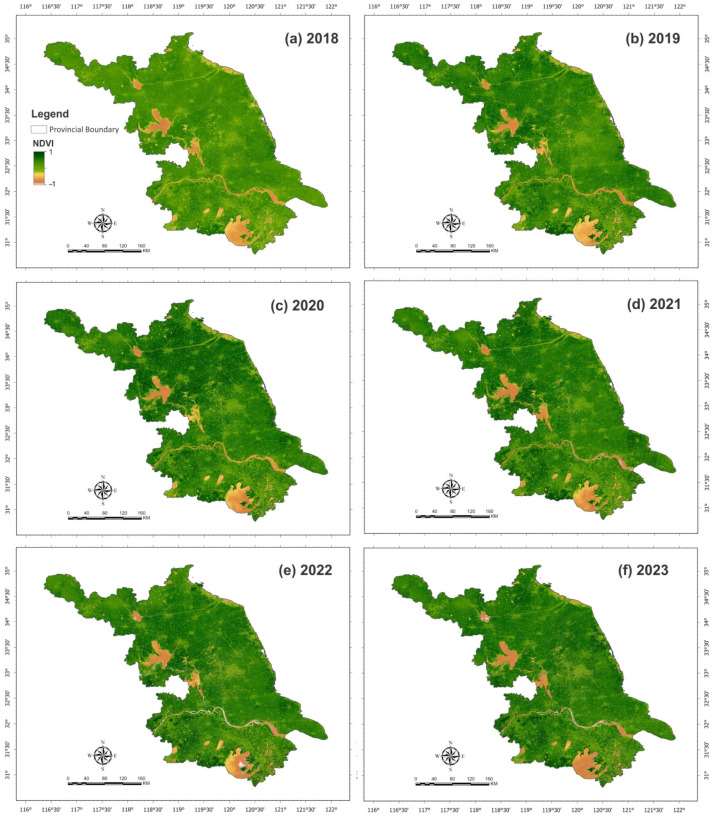
Spatial distribution of the Normalized Difference Vegetation Index (NDVI) across the study area during 2018–2023. Panels (**a**–**f**) present annual NDVI maps for 2018, 2019, 2020, 2021, 2022, and 2023, respectively, illustrating interannual variations in vegetation vigor and crop growth conditions.

The spatial–temporal distribution pattern of NDVI maps of Jiangsu Province from 2018 to 2023 is demonstrated in Figure 1. However, NDVI maps showed a light green color in 2018, indicating lower vegetation, while the dark green color seen in the northern region of province in 2019 and 2020 shows healthy vegetation. The NDVI maps also highlight the highest healthy vegetation in the southern region of Jiangsu Province in 2022 and 2023.

**Figure 2:**
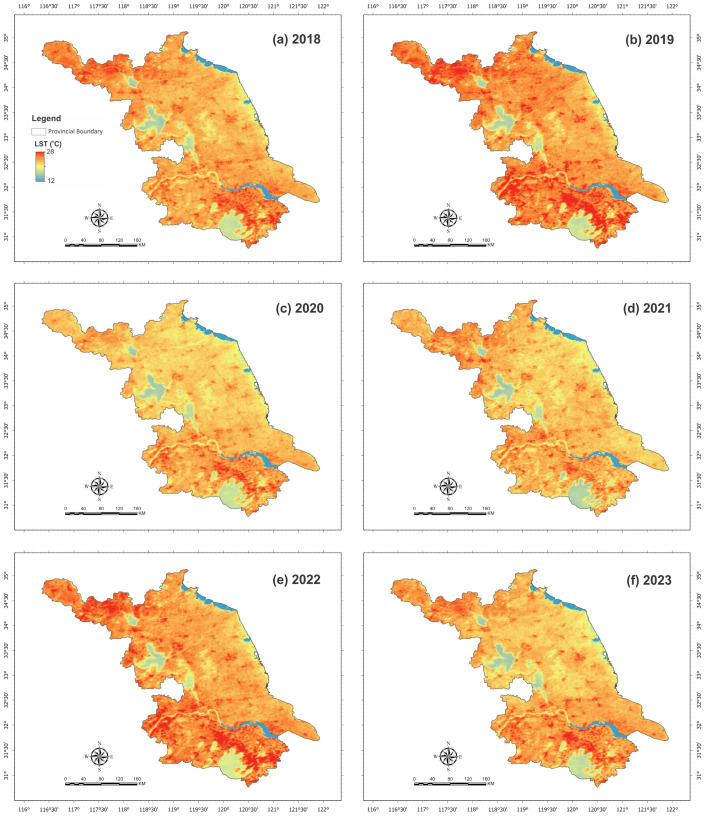
Spatial distribution of land surface temperature (LST) across study area, for the period 2018–2023. Panels (**a**–**f**) show annual LST maps for 2018, 2019, 2020, 2021, 2022, and 2023, respectively, illustrating interannual spatial variability and temperature patterns.

Figure 2 presents the LST-generated maps of Jiangsu Province from 2018 to 2023. The dark red color indicated a higher range of LST, and the light red color indicated the lower value of LST. During the six-year study period from 2018 to 2023, the western, northern and southern parts of Jiangsu Province observed the maximum LST range in 2019 and 2022. There might be several uncertain factors that might influence the LST range of Jiangsu Province in the studied year, such as lower precipitation, wind, sunlight, and water content.

### 2.2. Time Series Analysis of NDVI and LST

Overall minimum and maximum NDVI ranges fluctuate between −1 and 1.0 throughout the study period shown in Table 1. However, the lowest NDVI values, −0.47 was recorded in 2018, with a mean value of 0.30. In contrast, the higher mean values 0.42 were observed in 2020 and 2023, respectively. The mean NDVI values for 2021 and 2022 were slightly lower at 0.41 and 0.40. The average NDVI values from 2018 to 2023 show that vegetation became greener over the course of the study. For the year 2018, the minimum and maximum LST range was between 12.5 °C and 30.7 °C, with an average mean value of 23.3 °C (Table 1). In the year 2019, the LST range of Jiangsu Province was 7.4 °C to 32.9 °C, with a mean value of 24.3 °C. On the other hand, in 2020, and 2021, the mean annual LST values decreased by 22.9 and 22.8, respectively, as compared to 2019. From 2018 to 2023, mean temperature values indicate a slightly increasing trend.

### 2.3. Single-Season Rice Cropping Area Estimation

**Figure 3:**
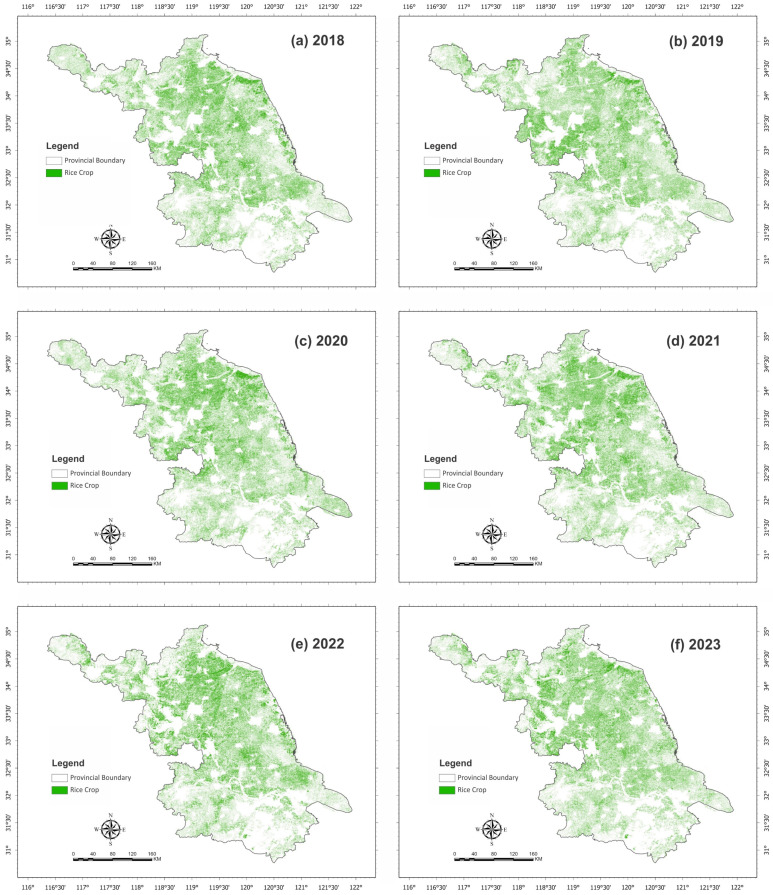
Spatial distribution of single-season rice (SSR) cropping areas in Jiangsu Province, during the rice growing season (April–September) for the period 2018–2023. Panels (**a**–**f**) represent SSR maps for 2018, 2019, 2020, 2021, 2022, and 2023, respectively, illustrating year-to-year spatial variability and persistence of rice cultivation patterns across the province.

The single-season rice cropping distribution area images acquired from April to September of the years 2018 to 2023 are presented in Figure 3. Sentinel 2A-generated maps indicate that the SSR cropping areas remained stable during the 2020 to 2023 study period, but eastern and southern rice areas indicate minor changes. The northern part of the province had a larger SSR cropping pattern compared to the southern part.

The combined confusion matrix and accuracy summary metrics (Table 2) demonstrate consistently reliable classification performance across the 2018–2023 period. Overall classification accuracy ranges from 77.33% to 93.55% with a Kappa coefficient from 0.55 to 0.87, reflecting an improvement from moderate to substantial agreement between the predicted map and field observations. The rice class achieved a producer accuracy ranging from 72.67% to 95.88% and a user accuracy of 80.15% to 95.88%, indicating strong reliability in correctly identifying rice fields across all years. For the non-rice class, the producer accuracy was 80.00% to 92.79%, while the user accuracy varied between 75.00% and 85.11%, indicating commission errors primarily associated with spectrally similar vegetation types such as other crops and mixed vegetated surfaces. Corresponding F1-scores further supported these findings, with ranges for the rice class from 0.76 to 0.96 and for the non-rice class from 0.78 to 0.88 indicating strong performance for rice detection and moderate performance for non-rice discrimination.

Furthermore, detailed year-wise confusion matrix-based validation is provided in Table S1, while interannual changes in accuracy metrics are summarized in Table S2, which indicates no systematic or linear decline in classification performance across the study period from 2018 to 2023. A brief and minor decrease in overall accuracy (ΔOA = −0.33%) and Kappa coefficient (Δκ = −0.01) was observed between 2019 and 2020; however, classification accuracy improved in subsequent years. Over the full 2018–2023 period, overall accuracy increased by +16.22%, and the Kappa coefficient increased by +0.32, demonstrating the stable interannual performance of the random forest model.

### 2.4. Numerical Results of Single-Season Rice Cropping Pattern

**Figure 4:**
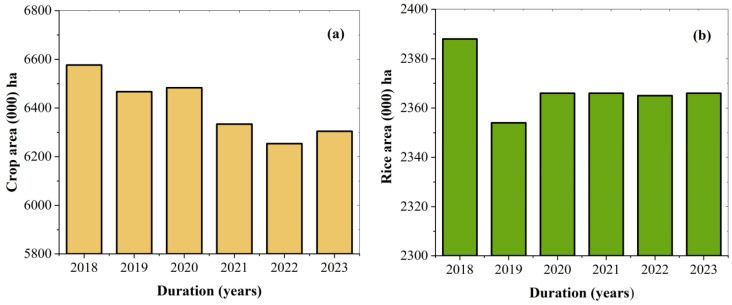
Temporal variation in cultivated land area of study area, from 2018 to 2023. (**a**) shows the total cultivated crop area (000 ha), while (**b**) presents the single-season rice cropping area (000 ha) on the provincial scale. The figure highlights contrasting trends between overall cropland reduction and the relative stability of rice cultivation during the study period.

According to the numerical data, results of the SSR cropping pattern showed that the highest reduction was 1.42% in 2019, and a minor decreasing trend of 0.94% was observed in 2022 and 2023 compared to 2018. Additionally, from 2020 through 2023, SSR remained stable. However, the total crop cultivated area decreased in 2019 compared to 2018, as shown in Figure 4. In contrast, total crop cultivated areas demonstrated the most significant decrease, i.e., 4.91% and 4.12% in 2022 and 2023, respectively, compared to 2018. On the other hand, the total cropping area showed a higher reduction, while the rice cropping area was slightly decreased from 2018 to 2023 in Jiangsu Province. Furthermore, key findings and model accuracy were compared with previous studies shown in Table 3. In the current research study, it was noticed that from 2018 to 2023, the rice area decreased and the RF model overall accuracy ranged from 77.33% to 93.55%.

### 2.5. Classification of LULC

**Figure 5:**
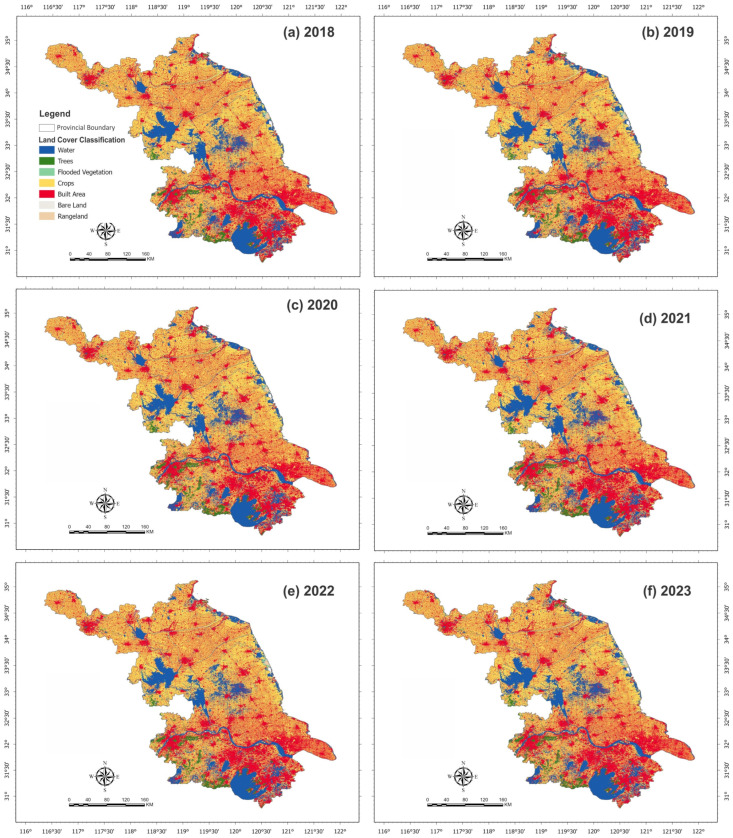
Spatial distribution of land use and land cover (LULC) classes across the Jiangsu Province, China, derived from annual remote sensing classification for the period 2018–2023. (**a**–**f**) illustrate LULC maps for 2018, 2019, 2020, 2021, 2022, and 2023, respectively. These maps highlight the spatial and temporal evolution of major land cover categories, including cropland, built-up areas, water bodies, and other land cover types, providing a basis for assessing land use dynamics and long-term changes across the province.

LULC classified maps of Jiangsu Province from 2018 to 2023, as shown in Figure 5. The LULC is categorized into seven classes (i.e., water, trees, flooded vegetation, crops, built-up areas, rangeland, and bare land). The province shows a dominance of yellowish-orange areas, indicating crops, but there are large red areas, especially in the eastern and southern regions, representing built-up areas. Particularly in the southern region, a narrow belt of dark green represents tree cover, while light green indicates areas dominated by flooded vegetation. The southern part of the province continues to show healthier vegetation cover (dark green) compared to previous years. However, the LULC classification maps show that the southern part of the Jiangsu region contains most of the built-up areas. The province continuously showed the development of urbanization, particularly in southern and central regions, as indicated by the red areas.

### 2.6. Assessment of LULC Numerical Data

Based on numerical data of land cover groups (such as water, trees, crops, built-up areas, rangeland, flooded vegetation, and bare land) there was an increasing and decreasing trend from 2018 to 2023, as shown in Table 4. The highest water area increased by 71.4 thousand hectares from 2019 to 2022. However, the LULC classification maps show that most of the built-up areas in the southern part of the Jiangsu region increased in the following pattern: 3879.2, 3966.8, and 3944.3 (000) ha in 2021, 2022, and 2023, respectively. The highest crops covered land was detected at 6577.3 ha in 2018, and the lowest crop area was seen in 2022 at 6254.20 ha. In 2022, the development of built-up areas to meet the increasing demand for urbanization led to decreased crop areas. However, rangeland and flooded vegetation-covered areas increased in 2023 by 61.4% and 163.2% as compared to 2018.

### 2.7. LULC Trend and Its Modifications

During the study period from 2018 to 2023, LULC changes exhibited diverse trends across different categories, as shown in Table 5. Water bodies expanded by approximately 0.21%, while tree-covered areas increased by 5.60% of the total area. The increasing number of tree-covered areas may have contributed to the reforestation activities. In contrast, crop cover decreased by 4.13% throughout the study period. On the other hand, in Jiangsu Province, the built area increased by 4.60% due to continued infrastructure development and urbanization. However, rangeland and flooded vegetation showed a higher significant increasing trend, i.e., 61.4% and 163.2%, throughout the study period. This rising trend is due to modified land management techniques, greater precipitation, and altered hydrological conditions. While barren land decreased by 24.9% from 2018 to 2023. These trends highlight the impact of both natural processes and human activity on the landscape, revealing significant changes in land use and cover dynamics. Spatial comparison of SSR maps with LULC transition data indicates that regions exhibiting SSR decline correspond closely with areas where cropland was converted to built-up land between 2018 and 2023. Southern and eastern Jiangsu show concurrent decreases in rice-covered areas and increases in built-up surfaces, suggesting that urban expansion is a primary driver of SSR loss.

### 2.8. Conservation of LULC Classes

The temporal conversation distribution of LULC classes, such as water, trees, crops, built-up areas, rangeland, flooded vegetation, and bare land from 2018 to 2023 is shown in Table 6. The analysis reveals that all LULC classes transitioned into other categories, largely driven by climatic variations. The maximum water bodies of 38,244 ha were converted into built areas, while the highest trees covered an area by 23,740 ha which was transformed into crop cover areas. Over the period from 2018 to 2023, the land use covered the classification of the highest crop areas in Jiangsu Province that were transferred into built-up areas, i.e., 491,567 ha, indicating a significant reduction in cropland combined with a consistent increase in built areas, indicating extensive urbanization and infrastructure development. Other categories, such as rangeland and barren lands, were significantly transferred into built areas. Flooded vegetation was the highest-transforming into water bodies, by 2275 ha. Similarly, the area with the highest water cover transitioned into barren land, while for bare land, the highest part converted into built area was 2203 ha. These findings clearly indicate that most LULC classes, such as water, crops, rangeland and bare land, are converted into built areas.

## 3. Discussion

The present study focuses on recent changes in the single-season rice (SSR) cropping area distribution pattern for food security using Sentinel-2 satellite data. Rice is one of the most cultivated crops worldwide and plays a critical role in ensuring global food security, particularly in densely populated regions. For estimating rice cropping area, scientists adopt different machine learning techniques and algorithms for accurate mapping of rice areas, utilizing both moderate- and high-resolution imagery across varying spatial scales, from small to large areas [34]. Ground-based approaches are time-consuming and labor-intensive to acquire high precision and a large volume of training samples [35,36]. But the ground provides reliable and accurate information as compared to assumption or only depending on remote sensing data. Therefore, the current study utilized a dual approach of ground sampling and remote sensing data for estimation of SSR. Results of spatiotemporal analysis revealed that the rice cultivation area decreased by 1.42% in 2019 compared to 2018, with most of the changes in rice cropping area observed in the eastern and southern regions of the province. Furthermore, the results indicate that the rice cover area remained relatively unchanged during the period 2020–2023. Data with high spatial and temporal resolution is usually advantageous because it provides more precise yield forecasts by capturing minute changes in crop growth and how they respond to environmental changes throughout the growing season. Previous research reported that rice production increased in Jiangsu Province from 2017 to 2022. Monitoring crop production has a direct effect on national and global economies and plays a significant role in food security. This study creates a possible autoregressive integrated moving average (ARIMA) model that can estimate the past (2010 to 2022) and future trends (2023 to 2035) for cultivated cropland and fertilizer consumption and their effects on rice and wheat production. The study results demonstrated past and future trends for different variables such as cultivated cropland, fertilizer consumption and rice and wheat production over time. Based on the ARIMA model analysis, a 2.4% and 113% total reduction in cropland and fertilizer consumption over the next 13 years, respectively, was predicted. Over the next 13 years, the production of major crops, specifically rice and wheat, is expected to increase by 12.4% and 25.9%, respectively. However, the multiple linear regression model showed a significant change for dependent variables such as cropland and fertilizer consumption, with R^2^ values of 61% and 74%, respectively, for rice and wheat production. The predictive results from the ARIMA model analysis possibly showed an increasing trend for estimating crop yields, with a minor change in cultivated cropland and highly decreased fertilizer consumption. These results highlight that higher crop production can be achieved with less cropland and with minor fertilizer input [33]. This study is based on autoregressive integrated moving average models. Additionally, it is predicted that this trend will continue by 2035. Furthermore, the results of the accuracy assessment in Table 2 show overall accuracy ranges from 77.33% to 93.55% with a Kappa coefficient of 0.55 to 0.87. Comparable with a previous study’s RF model for rice estimation, similar accuracy levels, generally ranging between 80% and 90%, were observed. A research study reported OA by 0.97 and Kappa coefficients greater than 0.95 [37]. The lower precision observed for the non-rice class reflects commission errors caused by spectral overlap between rice and other vegetation types; however, this limitation does not substantially affect the stability of relative interannual rice area trends, which are the primary focus of this study.

In the previous study described by [38], they generated three crop images, including rice, using a random forest technique from 2017 to 2020, and obtained accurate information for both producers and users of rice of >90%, except for the user precision in 2017 (87%). The results of the current study fall well within the expected performance range of RF-based rice classification and demonstrate consistent agreement with findings from previous remote sensing studies. The combined investigation of both parameters with the NDVI and LST to determine its impact on rice cropping patterns. The NDVI distribution derived from the Sentinel-2 satellite efficiently monitors vegetation health, primarily noted in 2019, and these estimates support the crop growth patterns observed in Jiangsu Province, while LST has both positive and negative impacts on agricultural production. Changes in climate conditions can significantly impact crop production, but the effects of annual climate variations are unpredictable, with different regions responding differently to climate change [39]. The present results demonstrate that a higher range of LST can significantly influence rice cultivation land. Extreme weather conditions could negatively impact agriculture production capacity, and agricultural disasters could reduce edible crop production [40]. Additionally, NDVI distinguishes vegetative cover areas from other land use and land cover classification groups [40]. It represents different types of live green land cover, such as crops, agroforestry, and thin and strong vegetation. The minimum and maximum range of NDVI is −1 to 1, which represents the vegetation density and non-vegetation coverage of the study area. The highest NDVI values showed that growing struggles have brought about improvements to the vegetation index and better agricultural practices [41]. The NDVI can assist in the assessment of vegetation conditions and coverage, enabling decision makers to undertake equitable and sustainable land management to restore vegetation cover. Furthermore, previous studies have found that higher vegetation density can help to maintain soil moisture and a reduction in LST [42]. The data also suggests that LST is expected to rise in 2019 and 2022 (Table 1). There are many uncontrollable factors that could raise the LST, including climate change, illumination, and low precipitation. The study area is one of most developed provinces in China. Reducing LST while preserving vegetation and improving crop patterns is key to sustainable development in the region. It has become a serious target for maintaining cropping patterns to improve sustainable development for the future due to rapid climate changes.

Although the RF classifier was trained using field data from a single year (2023), it was applied to multiple years using identical seasonal windows and predictor features to enhance temporal comparability. Nevertheless, interannual variability in crop management, phenology, and background land cover may influence classification performance. As a result, the derived SSR maps are most suitable for analyzing relative interannual trends rather than precise absolute area estimates for individual years. Future studies should incorporate multi-year field samples or temporally adaptive training strategies to further improve cross-year generalization.

The spatiotemporal analysis of LULC to understand the impact of human activities on urban planning, which influences sustainable environmental [43]. The land use classification results showed water bodies and tree-covered areas increased by 0.21% and 5.60% throughout the study period. However, climate change has led to changes in precipitation patterns, and this increased precipitation has improved water bodies such as lakes and reservoirs; more surface water accumulates in rivers. Changes in water resources have the potential to maintain ecosystem stability, enhance ecosystem quality, and stimulate afforestation. In addition, for the development of delicate green vegetation environments, the Chinese government has continually implemented conservation projects for environment protection such as planting trees, recovering vegetation, and returning farmland to forests [10]. According to RS established maps and statistical data, the crop cultivation area has decreased by 4.13% in recent decades, whereas the built-up area has increased by 4.60% in Jiangsu Province. This rapid urbanization has resulted in a significant decrease in agricultural land. This reduction in crop areas emphasizes how essential it is to continuously monitor and analyses LULC elements in order to have significant implications for agricultural productivity and food security, better understand the effects of urban growth on agricultural land, and guide sustainable urban design [44]. NDVI and LST were not used as predictive variables for SSR mapping, but rather as complementary indicators to contextualize rice area stability and decline under changing environmental and urban conditions.

There is a need to integrate LULC data into regional planning, which is necessary in order to prevent negative effects on the environment and food security as urban areas grow and the protection of agricultural and natural landscapes becomes more important [45]. A 24% reduction change was observed in barren land from 2018 to 2023. Bare land denotes a change that results in a reduced vegetative cover, potentially due to factors such as land degradation and deforestation. A reduced proportion of bare land could signify effective reforestation or the resurgence of natural vegetation. Landscape trends in bare soil areas can reveal long-term enhancements in agricultural land and environmental issues [41]. The LULC images are major data sources for agricultural land management and urban planning. An earlier study emphasizes the need to combine different types of RS data and use their unique abilities to produce accurate LULC maps [4]. The current study examined the LULC conversion maps from 2018 and 2023, utilizing advanced remote sensing methods, and found that the incorporation of optical and radar Sentinel 2A data significantly improved the precision of LULC classification. A former study that supports the present research demonstrated that combining of these data sources provides additional information, significantly improving the reliability of LULC mapping [46]. The LULC category classes, such as water bodies, trees, and crop-covered land areas, were significantly transferred into the built area. These changes contribute to the overall increase in urban areas within the region, potentially leading to higher LST and less agricultural land [47]. This pattern is largely due to the limited use of innovative agricultural technologies between 2018 and 2023 and the absence of organized cropping patterns, both of which impacted agricultural progress. Current findings indicate that water bodies experience the most significant changes in flooded vegetation. In the dry and wet seasons, the flooded vegetation process plays a crucial role in the physio-chemical properties of the soil and vegetation growth further; in the rainy and wet seasons, most of the water level and the water area increase, whereby most of the vegetation is submerged by water [48]. This study does not aim to construct a formal driver attribution model (e.g., MK trend tests or GeoDetector analysis), but rather to provide an integrated, spatially explicit assessment of SSR dynamics and their co-occurrence with land use and environmental changes. Quantitative driver attribution is identified as a priority for future research. Furthermore, the objective of this study is not to introduce new remote sensing algorithms, but to demonstrate how established datasets can be integrated to generate SSR-specific evidence relevant to regional land use planning and food security assessment.

### Temporal Transferability, Limitations, and Future Directions

Overall, although the random forest classifier was trained using field reference data from 2023, its temporal transferability was quantitatively assessed across the period (2018–2023) using independent validation datasets (Table 2 and Tables S1 and S2). Key results indicate stable temporal robustness rather than progressive degradation, with overall accuracy values ranging from 77.33% in 2018 to 93.55% in 2023 and Kappa coefficients increasing from 0.55 to 0.87, reflecting an improvement from moderate to substantial agreement. The rice class performance remained consistently strong across years (PA: 72.67–95.88%, UA: 80.15–95.88%, F1-score: 0.76–0.96), and interannual analysis revealed no systematic or linear decline, aside from a brief and minor reduction between 2019 and 2020 (ΔOA = −0.33%; ΔKappa = −0.01), followed by continued improvement (Table S2).

Interannual variability is attributed to factors inherent to multi-temporal RS, including shifts in rice phenological timing, climatic variability (as indicated by land surface temperature variations; Table 1), evolving agricultural practices, and minor radiometric differences in Sentinel-2 imagery. However, all evaluated years maintained overall accuracy above 77%, exceedingly commonly accepted operational thresholds for land cover mapping reliability. Some limitations remain: validation samples for 2018–2022 were derived through visual interpretation rather than contemporaneous field surveys, introducing potential interpretation uncertainty (Table S1), and the non-rice class exhibited comparatively lower UA (75.00–85.11%) due to spectral confusion with other vegetation types. Future work could address these limitations by incorporating multi-year training datasets, applying temporal domain adaptation techniques, and integrating Sentinel-1 SAR data to enhance detection during cloud-prone and flooded transplanting stages. Despite these constraints, the proposed framework grounded in established datasets, transparent validation, and multi-year performance assessment provides scientifically defensible rice area estimates suitable for regional land use planning and food security assessment in Jiangsu Province.

## 4. Material Methods

### 4.1. Description of Study Area

**Figure 6:**
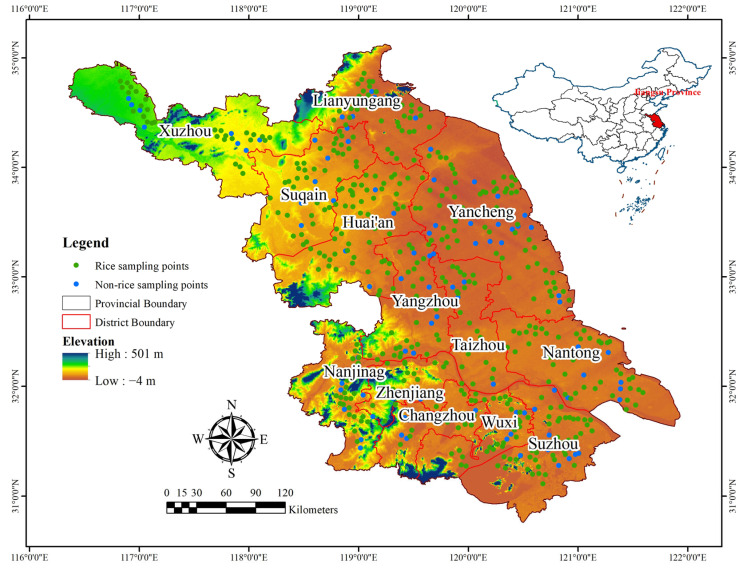
Geographic location of the study area in Jiangsu Province (eastern China). The provincial area boundary is outlined in black, and internal red lines indicate administrative subdivisions within the province. The background represents elevation from a digital elevation model (DEM), ranging from −4 m (low-lying plains; warm colors) to 501 m (higher terrain; cool colors). Green points mark rice sampling locations, while blue points mark non-rice sampling locations. The inset map highlights the position of Jiangsu Province within China. Geographic coordinates are shown in longitude/latitude (°E, °N), with a north arrow and scale bar (km) for spatial reference.

Jiangsu Province, located on the Yangtze River in eastern China, is one of the most economically established regions of the country, situated between east longitude 116°21″ to 121°54″ and north latitude 30°46″ to 35°08″, as shown in Figure 6. The average mean temperature is 15.9 °C, and the average mean precipitation fluctuates between 781 and 1382 mm. The total area of the province is 102,600 km^2^, with a typical rainy season from a hot–temperate zone to a northern subtropical zone. The gross domestic product (GDP) of this region is expected to reach CNY 12.29 trillion, accounting for 10.2% of the national GDP in 2022, and approximately 85.5 million people were recorded in 2022 [49]. Jiangsu Province is a major grain producing region of the country, producing wheat, rice, maize, soybeans, rapeseed, and peanuts. The cultivated land area of Jiangsu Province is 45,800 km^2^, representing 3.4% of China’s total cultivated land area [50].

### 4.2. Description of Flowchart

**Figure 7:**
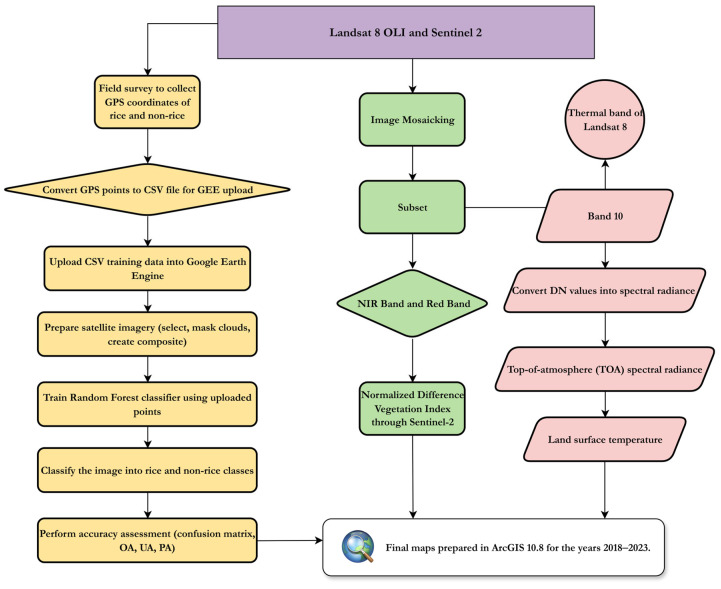
Flowchart of methodology.

Figure 7 describes the flowchart of the study, which presents the main study objectives. This study used different satellite data, such as Landsat-8 Operational Land Image (OLI)/Thermal Infra-Red Sensor (TIRS) and Sentinel 2A. Sentinel-2 data with ML algorithms was also used to investigate the NDVI, single-season rice cropping area, and LULC classification. Furthermore, Landsat-8 satellite data is used for LST analysis. This evaluation supports the single-season rice cultivation pattern, with the aim of further determining the economic and sustainable development of the study area. We have taken several steps in this process and provided more details in Section 2.3.

### 4.3. Data Collection and Processing

Single-season rice (SSR) mapping was conducted using multispectral surface reflectance imagery from the European Space Agency’s (ESA) Sentinel 2A satellite (Level 2A), accessed and processed through Google Earth Engine (GEE). The Sentinel-2 satellite provides a total of 13 spectral bands with high radiometric quality, enabling detailed vegetation and surface water monitoring. For NDVI, mapping bands 4 (665 nm) and 8 (842 nm) are used to characterize crop greenness and rice phenology. LST maps were generated from Landsat 8 OLI/TIRS satellite data acquired and analyzed through GEE [51]. All Sentinel-2 satellite scenes acquired during the rice growing season (April–September) from 2018 to 2023 were first subjected to cloud and cirrus masking using the QA60 quality assessment band to minimize atmospheric contamination. The cloud-masked imagery was then temporally aggregated into 15-day median composites, which effectively reduce residual cloud effects and short-term noise while preserving crop phenological signals. These composites were subsequently combined to generate annual predictor layers, ensuring temporal consistency across all study years. The predictor feature set used for random forest classification was constructed from selected Sentinel-2 satellite spectral bands (B2, B3, B4, B8, B11, and B12) and six vegetation and water-related spectral indices: NDVI, Enhanced Vegetation Index, Land Surface Water Index, Normalized Difference Water Index, Green Chlorophyll Vegetation Index, and Soil-Adjusted Vegetation Index. The selection of indices is based on their proven effectiveness in discriminating paddy rice by capturing vegetation vigor, soil background effects, chlorophyll concentration, and surface water presence during transplanting stages.

Georeferenced (ground truthing) data were collected through field surveys during the 2023 rice growing season (April–September) with the help of QField mobile application with GPS positioning. A total of 1700 rice samples and 470 non-rice samples were collected across Jiangsu Province to ensure representative spatial coverage. These samples were imported into GEE and used for classifier training and validation. A random forest classifier was implemented in GEE using 500 decision trees and a bagging fraction of 0.7, while other parameters were kept at default values. The model was trained using the 2023 ground reference data and subsequently applied to all study years (2018–2023) using the same predictor feature set to maintain methodological consistency. Post-classification processing included masking non-agricultural areas using cropland information derived from the ESRI 10 m land cover product to reduce commission errors. The final outputs consist of spatially consistent annual SSR distribution maps for Jiangsu Province.

### 4.4. NDVI Retrieval

NDVI is mainly examined to evaluate the healthy vegetation that can reflect the vegetation thickness of the land. It is mostly derived from the RED and near-infrared bands (NIR) of satellite imaginaries [52]. NDVI values range from −1 to 1, which specifies the vegetation condition. A number near 1 indicates high green vegetation, while values between 0.2 and 0.9 indicate thick, healthy vegetation. However, understanding the calculated NDVI values is helpful in identifying the vegetation, as you can use Equation (1) to obtain NDVI values:

(1) NDVI = (NIR − RED)/(NIR + RED)

### 4.5. Land Surface Temperature

LST is an important feature to understand how different land cover classification categories can reflect the surface temperature, which is crucial for climate change effects. Landsat imagery follows these critical steps to ensure accurate temperature retrieval. The first step involves converting the thermal band data into a numeric number (DN) and then converting it into units of spectral radiation at the Top Of Atmosphere (TOA) [53]. Therefore, to calculate the thermal band 10 on GEE, use Equation (2). After TOA conversion, apply spectral radiation using Equation (3) to calculate the brightness temperature (BT). In addition, the NDVI can be calculated for differentiating between vegetation cover (Equation (4)):

(2) L_λ_ = (M_L_ × Q_Cal_) + A_L_

where

L_λ_: Spectral radiation TOA. M_L_: 10 RADIANCE_MULT_BAND. A_L_: Radiance_Add_Band_10;

Q_Cal_: Quantized and calibrated DN values.

(3) BT = (K_2_/(ln(K_1_/L_λ_) + 1)) − 273.15

where

BT shows brightness of temperature, K_2_ shows calibration constant 2 and K_1_ represents the calibration constant;

L_λ_: TOA Spectral Radiance.

The calculation of NDVI based on Landsat 8 is essentially applied to distinguish vegetation cover as shown in Equation (4):

(4) $$NDVI=\left(NIR-RED\right)/(NIR+RED)$$

where Pv is the percentage of NDVI pattern, which can be calculated through following Equation (5):

(5) PV = Square (NDVI − NDVImin)/(NDVImax − NDVImin)^2^

Land surface emissivity (ε) is an important factor in assessing LST. The ε is calculated using Equation (6); Refs. [36,54] describe the LST using Equation (7):

(6) ε = 0.004 × PV + 0.986

(7) LST = BT/1 + (λ BT/ρ) × ln(ε))

where BT indicts brightness temperature, ρ represents 1.438 × 10^−2^ mK, and E shows land surface emissivity.

### 4.6. Single-Season Rice Area Estimation and Validation

Ground truthing data for rice and non-rice classes were collected during the rice growing season from April to September in 2023. During the field visit, the QField mobile application, which enabled GPS activation and direct recording of geographic coordinates, was used. In total, 1700 rice samples and 470 non-rice samples were collected across Jiangsu Province. The georeferenced field samples were divided into training and independent validation datasets using random approach to representative class distribution. The validation samples were reserved exclusively for accuracy assessment and error evaluation. Using the trained model, a rice mask for the 2023 season was generated. The same classification scheme was then applied to produce multi-year rice masks for 2018, 2019, 2020, 2021, and 2022, using Sentinel-2A imagery. The machine learning-based classification workflow enabled the derivation of consistent SSR cropping maps for Jiangsu Province across all selected years. The ground reference dataset was randomly divided into independent training (70%) and validation (30%) subsets to ensure unbiased accuracy assessment. All reported accuracy metrics were derived exclusively from the validation samples and were not used during model training. The final output of the classification is a binary rice mask, where rice pixels are assigned to a value of 1 and non-rice pixels a value of 0.

### 4.7. Temporal Transferability Assessment

To assess the interannual robustness and temporal transferability of the RF classifier trained using ground reference data of 2023, a temporal cross-validation strategy was implemented across multiple historical years from 2018 to 2022. Because field reference data were unavailable for the years 2018 to 2022, independent validation samples were generated through visual interpretation of multi-temporal satellite imagery, following established accuracy assessment protocols [55,56,57]. In total, 300 validation points (150 rice and 150 non-rice) for each historical year were selected with stratified random sampling, maintaining a spatial distribution comparable to the 2023 field survey. Rice and non-rice classes were interpreted based on Sentinel-2 NDVI time series profiles to confirm characteristic rice phenological patterns, including flooded transplanting, vegetative growth, reproductive development, senescence stages and Google Earth Pro historical high-resolution imagery acquired during the peak rice growing season (July–September).

To enhance labeling reliability, all samples’ points underwent independent interpretation by two experienced data analysts, and only high-confidence samples were retained for validation. Inter-interpreter agreement was quantified using Cohen’s Kappa (κ = 0.87), indicating strong consistency between interpreters. The RF model trained on 2023 field data was applied without retraining to Sentinel-2 imagery from each historical year using identical predictor variables, phenological windows, and preprocessing workflows. Standard accuracy metrics including overall accuracy (OA), Kappa coefficient, producer’s accuracy (PA), user’s accuracy (UA), and F1-scores were calculated for each year using the corresponding independent validation dataset to quantify temporal transferability and assess interannual model robustness.

### 4.8. LULC Groupings

LULC classification is one of the biggest challenges to generating complex land cover distribution information from Landsat 8 data [58]. Various studies, depending on the selected region and spatial resolution of the satellite images, have employed numerous classification strategies [4]. In the current study, we used the ESRI Land cover (10 m) product for LULC analysis; this product is derived from Sentinel-2 satellite imagery using the DL approach, with the overall accuracy of 86% reported by [42]. Land use/land cover (LULC) maps are foundational geospatial data products needed by analysts and decision makers across governments, civil society, industry, and finance to monitor global environmental change and measure risk to sustainable livelihoods and development. There is a strong need for high-level, automated geospatial analysis products that turn these pixels into actionable insights for non-geospatial experts. The Sentinel 2 satellites, first launched in mid-2015, are excellent candidates for LULC mapping due to their high spatial, spectral, and temporal resolution. Advances in deep learning and scalable cloud-based computing now provide the analysis capability required to unlock the value in global satellite imagery observations. Based on a novel, very large dataset of over 5 billion human-labeled Sentinel-2 pixels, we developed and deployed a deep learning segmentation model on Sentinel-2 data to create a global LULC map at 10 m resolution that achieves state-of-the-art accuracy and enables automated LULC mapping from time series observations. The LULC time series dataset is available in the GEE community catalog (https://gee-community-catalog.org/projects/S2TSLULC/ (accessed on 23 September 2024). This dataset provides annual LULC maps with high resolution from 2017 to 2023, covering the entire globe. In this land cover dataset, data are classified into nine distinct categories: water bodies, trees, flooded vegetation, crop areas, built-up area, bare ground, snow/ice, clouds, and rangeland. The water bodies classification covers all water bodies, e.g., lakes, ponds, reservoirs, wetlands, and rivers. Tree areas are covered with forest, woodland, and plantations. The crop grouping of agricultural lands without tree canopy. The built area classifies impervious surfaces including roads, parks, sidewalks, buildings, and airports. Rangeland areas are covered in standardized grasses with little to no taller vegetation and wild cereals and grasses with no obvious human plotting (i.e., natural meadows and fields). While flooded vegetation covers vegetation and plants that are fully or partially submerged in water due to flooding. Mostly covered with rock, soil, and desert sand, the bare land remains empty of vegetation all year round.

## 5. Conclusions

The present study investigated the spatiotemporal dynamics of single-season rice cropping area estimation in Jiangsu Province with ground reference data and a remote sensing approach. The remote sensing data highlights a significant reduction of 1.42% in rice cropping in 2019 as compared to 2018, while the total cultivated crop area significantly decreased by 4.13% from 2018 to 2023. The rice cropping area remained stable during the 2020–2023 period, while the total cultivated cropping area significantly decreased in the province, primarily due to rapid urbanization development. The expansion of the built area in the province has not affected the rice cropping area. But in the same duration, the total crop area decreased. However, in the years of 2019 and 2022, increasing LST may also have had effects on crop production. These results emphasize the significance of incorporating environmental monitoring into agricultural management strategies to respond to the negative impacts of climate change and urbanization, as evidenced by the high resolution of single-season rice maps. Temporal cross-validation across 2018–2023 confirmed the robustness of the random forest classification framework, with overall accuracy ranging from 77.33% to 93.55% and no systematic accuracy decline. The study demonstrates that single-year training, when combined with rigorous temporal validation, can produce reliable SSR maps for regional land use planning and food security assessment. The current study suggests that future research should focus on SSR at a finer spatial scale and provide detailed district- and city-level information, as this would be more helpful for improving food security.

## Figures and Tables

**Table 1:** Statistical minimum, maximum and mean values of land surface temperature (LST) and Normalized Difference Vegetation Index (NDVI) from 2018 to 2023 of Jiangsu Province.

Indices	Years	2018	2019	2020	2021	2022	2023
NDVI	Min	−0.47	−0.65	−0.65	−0.99	−1.0	−1.0
	Max	0.81	0.90	0.91	0.99	1.0	0.98
	Mean	0.30	0.39	0.42 ↑	0.41	0.40	0.42 ↑
LST	Min	12.5	7.40	14.4	8.5	14.1	10.7
	Max	30.7	32.9	30.4	30.2	32.6	30.3
	Mean	23.3	24.3 ↑	22.9	22.8	24.5 ↑	23.7

Note: The arrow shows an increasing trend in NDVI and LST (↑).

**Table 2:** Confusion matrix and accuracy matrix of rice and non-rice from 2018 to 2023.

Year	PA Rice (%)	PA Non-Rice (%)	UA Rice (%)	UA Non-Rice (%)	F1 Rice	F1 Non-Rice	OA	Kappa
2023	95.88	85.11	95.88	85.11	0.96	0.85	93.55	0.87
2022	82.00	92.79	91.79	83.73	0.87	0.88	87.33	0.75
2021	80.67	92.00	90.98	82.63	0.86	0.87	86.33	0.73
2020	78.67	91.33	90.08	81.07	0.84	0.86	85.00	0.70
2019	78.00	92.67	91.41	80.81	0.84	0.86	85.33	0.71
2018	72.67	82.00	80.15	75.00	0.76	0.78	77.33	0.55
Mean	84.68	89.82	90.05	81.71	0.86	0.85	85.98	0.72

**Table 3:** Compared the current research with previous studies of predictive model performance with key results.

Crop Type	Key Findings This Research	Findings from the Previous Literature	Agreement/Deviation	Likely Cause of Difference	Reference
Rice	RF classification at field/region scale	High pixel-level yield prediction accuracy using Sentinel-2 and ML (R^2^ up to 0.85); accuracy improves later in season	Complementary (not directly comparable)	Different objectives (area vs. yield), spatial scale (pixel vs. field), and target variable	[5]
Rice	RF model and optical time series analysis and improve class separability	Continental-scale rice mapping using Sentinel-1 VH SAR achieved OA > 70%; SAR revealed finer rice field details than optical-based products	Limited agreement	Differences in sensor type (SAR vs. optical), classification strategy	[22]
Rice	RF model classification for rice area estimation	Using RS and meteorological variables district-level yield prediction achieved high accuracy (92%; RMSE 328 kg/ha)	Complementary (not directly comparable)	Different target variable (yield vs. area), inclusion of meteorological drivers	[29]
Rice	Declining trend in recent years	Stable to increasing rice area	Deviation	Higher spatial resolution and updated datasets	[32]
Rice, Wheat	Similar production trends with spatial variability	Increased production with reduced inputs	Agreement	Consistent macro-scale behavior; local heterogeneity	[33]
Wheat	Comparable yield trends with moderate differences	RF + climate data, strong yield predictability	Limited agreement	Different time span and predictor configuration	[11]
Rice	Declining trends were observed from 2018 to 2023	Almost similar trends were seen (OA was 77.33% to 93.55)	Agreement	Field observation with remote sensing RF model predication	Current research

**Table 4:** Numerical data of land use–land cover (LULC) classification pattern in Jiangsu Province from 2018 to 2023.

LULC	Years					
	2018 (000) ha	2019 (000) ha	2020 (000) ha	2021 (000) ha	2022 (000) ha	2023 (000) ha
Water bodies	2001.5	1997.3	2036.0	2064.5 ↑	2068.7 ↑	2005.8
Trees	287.0 ↓	297.61	312.26 ↑	304.05	296.54	303.04
Crops	6577.3 ↑	6467.14	6483.6 ↑	6334.62	6254.2 ↓	6305.88
Built area	3770.8 ↓	3838.7	3800.8 ↓	3879.2	3966.8 ↑	3944.3 ↑
Rangeland	106.62	138.62	106.95 ↓	158.76	152.36	172.11 ↑
Flooded vegetation	8.2578 ↓	12.683	11.941	11.832	13.236	21.73 ↑
Bare land	5.634	5.209	4.656	4.176 ↓	5.310 ↑	4.226

Note: The arrows indicate increasing and decreasing trends in LULC (↑↓).

**Table 5:** Temporal changes in LULC based on Sentinel from 2018 to 2023.

Land Cover Type	Area (000) ha	Area (000) ha	TG Area (000) ha	TD Area 000 (ha)
	2018	2023	2018 to 2023	2018 to 2023
Water bodies	2001.5	2005.8	12,174.84	+0.21 ∆
Trees	287.0	303.04	1800.537	+5.60 ∆
Total crop area	6577.3	6305.88	38,422.89	−4.13 ∆
Built area	3770.8	3944.3	23,200.63	+4.60 ∆
Rangeland	106.62	172.11	835.4466	+61.4 ∆
Flooded vegetation	8.2578	21.73	79.68669	+163.2 ∆
Bare land	5.6345	4.2266	29.21375	−24.9 ∆

Note: ∆ represents the total variation of the LULC, TG (total geographical), and TD (total difference), while 000 ha means a thousand hectares.

**Table 6:** Numerical data indicates the temporal conversation changes in LULC classification from 2018 to 2023.

Land Use Land Cover Changes During 2018 to 2023		
Original Classes	Converted Class	Area (000) (ha)
Water	Trees	3556
Water	Crops	122,758
Water	Built area	38,244 ↑
Water	Rangeland	11,495
Water	Flooded vegetation	5373
Water	Bare ground	2240
Trees	Water	1987
Trees	Crops	23,740 ↑
Trees	Built area	12,987
Trees	Rangeland	12,720
Trees	Flooded vegetation	117
Trees	Bare ground	14
Crops	Water	222,214
Crops	Trees	42,450
Crops	Built area	491,567 ↑
Crops	Rangeland	57,603
Crops	Flooded vegetation	2146
Crops	Bare ground	614
Built area	Water	28,264
Built area	Trees	7908
Built area	Crops	280,375 ↑
Built area	Rangeland	25,474
Built area	Flooded vegetation	224
Built area	Bare ground	1338
Rangeland	Water	4603
Rangeland	Trees	8322
Rangeland	Crops	20,831
Rangeland	Built area	21,168 ↑
Rangeland	Flooded vegetation	2247
Rangeland	Bare ground	168
Flooded vegetation	Water	2275 ↑
Flooded vegetation	Trees	152
Flooded vegetation	Crops	860
Flooded vegetation	Built area	94
Flooded vegetation	Rangeland	1090
Flooded vegetation	Bare ground	2
Bare land	Water	744
Bare land	Trees	19
Bare land	Crops	825
Bare land	Built area	2203 ↑
Bare land	Flooded vegetation	21
Bare land	Rangeland	335

Note: The arrow indicates an increasing trend (↑).

## Data Availability

The data that support the findings of this study are available from the first author upon reasonable request.

## Supplementary Materials

The following supporting information can be downloaded at https://www.mdpi.com/article/10.3390/plants15050715/s1, Table S1: Confusion matrices and accuracy metrics for rice classification (2018–2023) using field-based and visual interpretation validation data.; Table S2: Interannual changes in class-wise and overall accuracy metrics for rice classification (2018–2023).
