# Supplementary material for: Estimating Rice Cropping Area and Analyzing Land Use and Land Cover Changes in Jiangsu Province Using Multispectral Satellite Imagery
Source: Plants (Basel). 2026 Feb 27;15(5):715. doi: 10.3390/plants15050715 (PMC12986608; doi:10.3390/plants15050715)
Supplement: Supplementary file 1 [file plants-15-00715-s001.zip › plants-4070673-supplementary.pdf]

Table S1 shows confusion matrices derived from reference rice and non-rice samples used to evaluate annual rice classification performance from 2018 to 2023. The 2023 classification was validated using field-collected reference data, while classifications from 2018 to 2022 were assessed using visual interpretation of high-resolution imagery. User's Accuracy (UA), Producer's Accuracy (PA), Overall Accuracy (OA), Kappa coefficient (κ), and F1-scores for rice and non-rice classes are reported to support assessment of classification reliability and temporal stability. Across the 2018–2023 assessment period, the OA ranged from 77.33% to 93.55%, with all years exceeding 77%, indicating reliable model performance throughout the study duration. The Kappa coefficient varied between 0.55 and 0.87, reflecting an improvement from moderate to substantial agreement over time. For the rice class, PA ranged from 72.67% to 95.88%, while UA ranged from 80.15% to 95.88%, demonstrating consistent discrimination of rice from non-rice areas across years. Temporal analysis showed no systematic (linear) accuracy decline; instead, OA exhibited an overall increasing trend from 2018 to 2023 (net gain of 16.22%, equivalent to ~3.24% per year) with minor interannual fluctuations. In addition, the F1-score for the rice class improved from 0.76 to 0.96, confirming robust and increasingly consistent class-level performance across the multi-year analysis.

Table S1. Confusion matrices and accuracy metrics for rice classification (2018–2023) using field-based and visual interpretation validation data

	2023 Baseline - Field Validation Data)		
	Rice	Non-Rice	UA (%)
Pred Rice	1630	70	95.88
Pred non-Rice	70	400	85.11
PA (%)	95.88	85.11	OA: 93.55%
	Kappa: 0.87	F1-Rice: 0.96	F1-non-Rice: 0.85
	2022 Visual Interpretation Validation)		
	Rice	Non-Rice	UA (%)
Pred Rice	123	11	91.79
Pred non-Rice	27	139	83.73
PA (%)	82.00	92.67	OA: 87.33%
	Kappa: 0.75	F1-Rice: 0.87	F1-non-Rice: 0.88
	2021 (Visual Interpretation Validation)		
	Rice	Non-Rice	UA (%)
Pred Rice	121	12	90.98
Pred non-Rice	29	138	82.63
PA (%)	80.67	92.00	OA: 86.33%
	Kappa: 0.73	F1-Rice: 0.86	F1-non-Rice: 0.87
	2020 (Visual Interpretation Validation)		
	Rice	Non-Rice	UA (%)
Pred Rice	118	13	90.08

Pred non-Rice	32	137	81.07
PA (%)	78.67	91.33	OA: 85.00%
	Kappa: 0.70	F1-Rice: 0.84	F1-non-Rice: 0.86
2019 (Visual Interpretation Validation)			
	Rice	Non-Rice	UA (%)
Pred Rice	117	11	91.41
Pred non-Rice	33	139	80.81
PA (%)	78.00	92.67	85.33
	Kappa: 0.71	F1-Rice: 0.84	F1-non-Rice: 0.86
2018 (Visual Interpretation Validation)			
	Rice	Non-Rice	UA (%)
Pred Rice	109	27	80.15
Pred non-Rice	41	123	75.00
PA (%)	72.67	82.00	77.33
	Kappa: 0.55	F1-Rice: 0.76	F1-non-Rice: 0.78

Table S2 summarizes the year-to-year and long-term interannual changes in Producer's Accuracy, User's Accuracy, Overall Accuracy, and Kappa coefficient for rice and non-rice classes between 2018 and 2023. Positive and negative values represent relative performance gains or losses between consecutive years. Results indicate predominantly positive interannual changes for rice classification, with only minor short-term fluctuations (e.g., a −0.33% change in OA and −0.01 change in κ between 2019 and 2020). Over the full 2018–2023 period, substantial net improvements are observed, including a +16.22% increase in OA, a +0.32 increase in Kappa, +23.21% improvement in rice PA, and +15.73% improvement in rice UA, demonstrating strong temporal stability and progressive enhancement of classification performance.

Table S2. Interannual changes in class-wise and overall accuracy metrics for rice classification (2018–2023)

**Period**	**ΔPA Rice (%)**	**ΔPA non-rice (%)**	**Δ UA Rice (%)**	**ΔUA non-rice (%)**	**ΔOA (%)**	**Δ Kappa**
2018-2019	+5.33	+10.67	+11.26	+5.81	+8.00	+0.16
2019-2020	+0.67	-1.34	-1.33	+0.26	-0.33	-0.01
2020-2021	+2.00	+0.67	+0.90	+1.56	+1.33	+0.03
2021-2022	+1.33	+0.79	+0.81	+1.10	+1.00	+0.02
2022-2023	+13.88	-7.68	+4.09	+1.38	+6.22	+0.12
2018-2023	+23.21	+3.11	+15.73	+10.11	+16.22	+0.32
